# Implant-supported crowns on maxillary laterals and canines—a long-term follow-up of aesthetics and function

**DOI:** 10.1007/s00784-023-05344-0

**Published:** 2023-11-09

**Authors:** Elnaz Sanatnama, Lena Frantz, Erik Ahlin, Julia Naoumova

**Affiliations:** 1 Public Dental Service, Region Västra Götaland, Gothenburg, Sweden; 2 Specialist Clinic for Orthodontics, Gothenburg, Public Dental Service, Region Västra Götaland, Gothenburg, Sweden; 3 Specialist Clinic for Orthodontics, Borås, Public Dental Service, Region Västra Götaland, Gothenburg, Sweden; 4 Department of Orthodontics, Institute of Odontology, Sahlgrenska Academy, University of Gothenburg, Gothenburg, Sweden

**Keywords:** Aesthetics, Implant, Lateral incisors, Canines, PROM, Orthodontists

## Abstract

**Objectives:**

To assess the long-term aesthetic and functional aspects of implant-supported crowns in lateral (ISC-L) and canine positions (ISC-C).

**Materials and methods:**

Thirty-two patients (14 males, 18 females, mean age: 23.1, SD:2.0) with an ISC-L or ISC-C participated in this prospective cohort study at baseline (T0) and in the long-term follow-up (T1, mean years: 11.1, SD: 1.0). Twenty-four patients (11 males, 13 females) participated in T1. Patient-reported outcomes (PROM) were rated using surveys with questions related to aesthetics and function. The colour of the implant crown and the buccal gingiva, the appearance of the papilla, periodontal health and temporomandibular disorder (TMD) outcomes were assessed in a clinical examination. The Mann-Whitney, Chi-square and Signed Rank tests were performed.

**Results:**

Patients with an ISC-L and ISC-C were equally satisfied with the crown shape and colour at T0 and T1. No differences in TMD outcomes were reported by the patients and no clinical signs of TMD were observed. At T1, ISC-C had more bleeding on probing and a three mm greater pocket depth than ISC-L (*p* = 0.03, *p* = 0.01, respectively). At T0, operators graded the crown colour of ISC-L as being too dark (35%) and ISC-C as being too light (40%). At T1, no difference was seen between the two groups regarding crown colour, gingiva colour and the papilla (*p* = 0.2749, *p* = 0.2099, *p* = 0.8053, respectively).

**Conclusions:**

The PROM and clinical examination show that ISC-L and ISC-C are equivalent with regard to aesthetics and function in the long term.

**Clinical relevance:**

Although ISC-L and ISC-C are aesthetically and functionally comparable in the long term, ISC-Cs are more likely to impact periodontal health.

**Supplementary Information:**

The online version contains supplementary material available at 10.1007/s00784-023-05344-0.

## Introduction

Absence of maxillary lateral incisors or canines is generally caused by hypodontia, which is often linked to genetics [1]. Other conditions, such as genetic disorders and syndromes, trauma, primary tooth retention or surgical removal due to impaction, may also increase the risk [2, 3]. Patients usually experience congenitally missing anterior teeth as both psychosocially and aesthetically deviating. The most common treatment options are either orthodontic space closure or replacing the missing tooth with an implant-supported crown (ISC) [4–6]. The choice of treatment is based on many factors: patient preferences, occlusion, available space in the dental arch and the apical bases, status of the neighbouring teeth, and the patient’s profile and growth pattern [7–9].

An orthodontic space closure is always preferred, for a number of reasons. Orthodontic treatment can begin and end at an early age, the treatment is both cost-effective and minimally invasive as biological structures are preserved and natural adaptation can occur over time, at the same time as alveolar bone height can be preserved [10, 11]. Moreover, it has been shown in a recent study that laypersons perceive orthodontic space closure more aesthetically acceptable [12]. However, in patients with large apical bases, space opening and placement of an ISC usually result in more aesthetically pleasing results. The disadvantage of dental implants is that it is a viable treatment option only after the completed growth phase during adolescence. Moreover, an implant may lead to an aesthetically unsatisfactory result by ending up in an infraocclusion position, the gingiva may become discoloured due to buccal bone loss, and the fixture may become visible due to gingival retraction [8, 13]. Placement of an ISC in the aesthetic zone often requires a multidisciplinary approach with the participation of orthodontists, periodontists, oral surgeons and prosthodontists to achieve an optimal aesthetic and functional result and to minimise complications.

When deciding which treatment will be the most suitable, the patient’s perception of teeth aesthetics is of great importance to the operator [1, 7, 14]. The perception of dental aesthetics can vary between individuals as well as between operators [9]. A person’s smile is perceived to be of great importance for communication and is strongly associated with facial attractiveness; hence, dental aesthetics play an important role in our social lives [15, 16].

Patients with agenesis of the lateral incisors generally have persisting deciduous canines and permanent canines that have erupted in the lateral position. An ISC is therefore placed at the canine or the lateral position if the option to distalise the canine is chosen. Previous studies have mostly focused on comparing treatment results after orthodontic space closure versus orthodontic space opening and implant installation [4, 13, 17]. Even though both treatment alternatives present advantages as well as disadvantages, the main conclusion from these studies is that orthodontic space closure, whenever it is possible, is advantageous over implant installation. However, studies are lacking—especially long-term follow-ups—comparing ISCs at the lateral incisor or the canine position.

Therefore, the purpose of this study was to evaluate the long-term result, both objectively and subjectively, after placement of an ISC at the lateral or canine position, with respect to both aesthetics and function.

The null hypothesis is that there is no difference between an ISC at the lateral position and/or canine position concerning aesthetics and function.

## Material and method

This study had a prospective cohort design and was conducted at the Specialist Clinic of Orthodontics in Borås, Public Dental Service, Region Västra Götaland, Sweden, between December 2021 and June 2023. The protocol of the study was approved by the research ethics committee of the Swedish Ethical Review Authority (reg. no: 2020- 01826).

### Subjects

Healthy patients who received a unilateral or bilateral ISC in the position of a maxillary lateral incisor or canine during 2003–2007 were included and categorised in two groups: “Implant-Supported Crown, Lateral (ISC-L),” and “Implant-Supported Crown, Canine (ISC-C).” Patients with syndromes or cleft lip and palate were excluded.

All patients had pre-prosthodontic orthodontic treatments performed by nine orthodontists. The surgical procedures to install an ISC were done by two oral and maxillofacial surgeon’s and the prosthetic treatment was executed by five prosthodontists. All patients were treated at the Specialist Clinics of Orthodontics, Oral and Maxillofacial Surgery and Prosthodontics in Borås, Public Dental Service, Region Västra Götaland, Sweden.

### Method

All patients had a clinical examination and completed two questionnaires at baseline (T0) and at the long-term follow-up appointment (T1). The examinations were carried out by two operators who were calibrated by examining the first five patients together.

### Patient-reported outcome (PROM)

Patient-reported outcome was assessed using a questionnaire with questions about the aesthetic and functional outcome. For the *aesthetic evaluation,* questions were asked about the shape and the colour of the ISC, the appearance of the gingiva, the symmetry between the right and left sides and whether the patient was satisfied with the aesthetic result and would recommend the treatment to others. In addition, a modified version of the “Eastman Esthetic index” was used to assess patient satisfaction with their general facial appearance with respect to the teeth and the general appearance of the teeth (Supplementary material A).

The *functional evaluation* was assessed using a temporomandibular disorder (TMD) health questionnaire, where patients were asked whether they experience headache, temporomandibular jaw joint (TMJ) sounds, tooth clenching or grinding, and to specify how often by choosing one of the five given statements (Supplementary material B).

### Clinical examination

The clinical examination included an objective assessment of the implant crown colour and the colour of the buccal gingiva using the rating scale: matches well, too dark, too light. In addition, the appearance of the mesial and distal papillae for each ISC was recorded using the papilla index: “absence of papilla,” “presence of 1/2 of the papilla height,” “papilla filling up the entire proximal space,” and “hyperplastic papilla.” The periodontal health of each implant tooth was evaluated by recording the presence or absence of plaque, bleeding on probing (BoP) and pocket depth < 3 mm at four locations on each tooth: mesial, distal, buccal, and palatal. In addition, buccal gingival retraction was recorded [18, 19].

Palpation of the jaw joints and jaw muscles was carried out to detect possible tenderness. In addition, patients’ dental records were reviewed for information about which fixture and ISC were used.

## Statistics

Data analysis was performed using the patient as the experimental unit. Descriptive statistics were calculated for demographic and clinical data at baseline and the long-term follow-up. The Mann-Whitney test was performed for the assessment of dependent continuous or ordinal data and the Chi-square test was performed to test the association between categorical variables. The Signed Rank Test was used to test the differences between baseline and follow-up for each group. All data analyses were performed using the Statistical Package for Social Sciences (SPSS Inc., version 16.0, Chicago, Illinois, USA) and the level of statistical significance in all analyses was set to 0.05.

Patient-rated questions with multiple choice options were dichotomised: For shape-related questions as satisfied = “good shape,” dissatisfied = “too pointed,” “too wide,” “too rounded,” “too thin” and “too short,” and no opinion = “other” and “no opinion”; for color-related questions as satisfied = “good colour,” dissatisfied = “too yellow,” “too grey,” “too dark,” and “too light,” and no opinion = “other” and “no opinion.” TMD-related questions were dichotomised into never, monthly = “*once or twice* a month,” weekly = “*once* a week,” and “several times a week,” and daily.

## Results

Thirty-two patients were included at T0. Of these, nineteen patients had an ISC-L, and thirteen patients had an ISC-C (Table 1). Two different types of fixtures were used: fixtures with bone-level external connection (Nobel) or fixtures with tissue-level internal connection (ITI). In 29 patients, the crown secured to the implant was made in ceramic and the rest in porcelain fused to metal. At T1 (mean years: 11.1, SD: 1.0), twenty-four patients attended the examination (Table 1). Reasons for dropping out were lack of interest (*n* = 4) or long travelling distance (*n* = 4).

**Table 1:** Patient characteristics at T0 (baseline) and T1 (long-term follow-up) in each group: ISC-L (Implant-supported crown – lateral) and ISC-C (Implant-supported crown – canine)

	T0 (*N* = 32)		T1 (*N* = 24)	
	ISC-L (*N* = 19) I = 27	ISC-C (*N* = 13) I = 17	ISC-L (*N* = 14) I = 20	ISC-C (N10) I = 12
Gender				
Male	10	4	8	3
Female	9	9	6	7
Age (mean, SD)	22.6, 2.0	23.8, 2.0	33.7, 2.2	34.9, 1.9
Absence of tooth				
Lateral	19	7	14	5
Canine	0	6	0	5
Reason				
Impacted	0	4	0	3
Aplasia	17	9	12	7
Resorption	2	0	2	0
Implant region				
Unilateral: Left side	1	2	0	2
Unilateral: Right side	9	6	8	5
Bilateral	9	5	6	3

*N* number of patients, *I* number of implants, *SD* standard deviation

### Patient-reported outcome (PROM)

#### Aesthetics

**Fig. 1:**
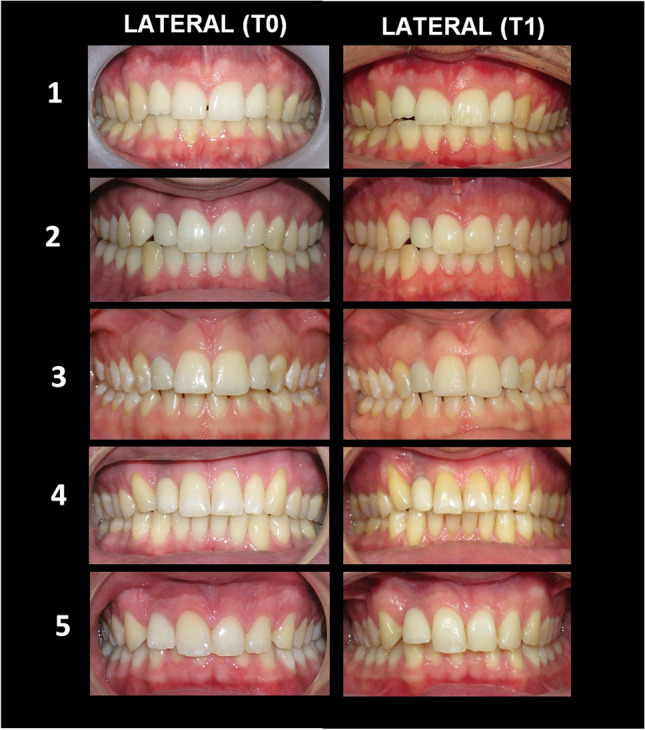
Intraoral photos of five cases with ISC-L (implant-supported crown – lateral) at T0 (baseline) and T1 (long-term follow-up). ISC-L on the right side is seen in cases #2,4,5 and bilateral ISC-L is seen in cases #1,3

**Fig. 2:**
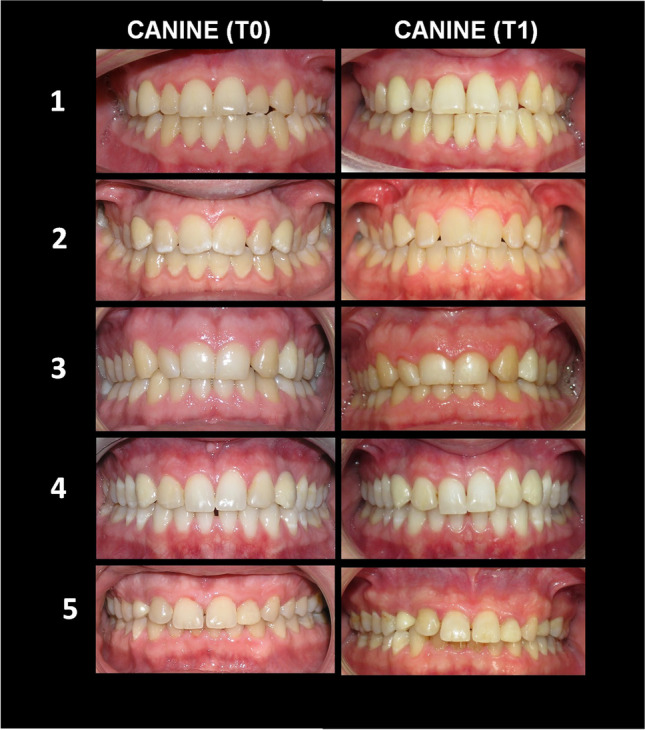
Intraoral photos of five cases with ISC-C (Implant-supported crown – canine) at T0 (baseline) and T1 (long-term follow-up). ISC-C on the right side is seen in cases #1, 5 and on the left side in case #3. Bilateral ISC-C is seen in cases #2,4

Patients with ISC-L and ISC-C were equally satisfied with the general aesthetic outcome at T0 and at T1 (Figs. 1–2). In both groups, most patients were satisfied with the aesthetic result of the implant and would consider going through the treatment again and recommend it to others (Table 2). Irrespective of the implant position, patients were similarly satisfied with the shape and colour of the ISC at T0 and T1 (Table 3). Patients with an ISC were more satisfied with the appearance of their teeth than with the general impression of the whole face at T0 than at T1 (*p* = 0.03 and *p* = <0.001). Similarly, patients with an ISC reported being more satisfied with the appearance of their teeth compared with friends of the same age at T0 than at T1 (*p* = 0.001 and *p* = 0.001) (Table 4).

**Table 2:** Patient-reported outcome of the treatment with ISC-L (Implant supported crown-lateral) and ISC-C (Implant supported crown-canine). “Complete group” includes all individuals who participated at T0 (baseline). “Subgroup follow-up” only includes individuals who participated both at T0 and in the long-term follow-up (T1)

Questions		Complete group			Subgroup follow-up						
		T0 (*N* = 32)			T0 (*N* = 24)		T1 (*N* = 24)			T0/T1	
		ISC-L	ISC-C	*P*-value	ISC-L	ISC-C	ISC-L	ISC-C	*P*-value	ISC-L *P*-value	ISC-C *P*-value
Are you satisfied with the aesthetic result?	Yes	17	13	0.50	14	10	12	10	0.49	1.00	-
	No	2	0		0	0	2	0			
Would you consider going through the treatment once again?	Yes	18	13	1.00	14	10	13	10	1.00	1.00	-
	No	1	0		0	0	1	0			
Would you recommend the treatment to others?	Yes	18	13	1.00	14	10	13	10	1.00	1.00	-
	No	1	0		0	0	1	0			
Do you experience the implant tooth as your own tooth?	Yes	12	10	0.47	10	7	10	8	1.00	0.72	1.00
	No	7	3		4	3	4	2			
Do you feel that it is more difficult to take care of the implant tooth than your own teeth?	Yes	4	5	0.43	2	3	7	5	1.00	0.14	0.69
	No	15	8		12	7	7	5

**Table 3:** Questions regarding aesthetic aspects of the crown shape and the colour of the ISC-L (Implant-supported crown-lateral) and ISC-C (Implant-supported crown-canine). “Complete group” includes all individuals who participated at T0 (baseline). “Subgroup follow-up” only includes individuals who participated both at T0 and in the long-term follow-up (T1)

Shape		Complete group			Subgroup follow-up						
		T0 (*N* = 32)			T0 (*N* = 24)		T1 (*N* = 24)			T0/T1	
		ISC-L	ISC-C	*P* value	ISC-L	ISC-C	ISC-L	ISC-C	*P* value	ISC-L *P* value	ISC-C *P* value
What do you think of the shape of the implant tooth?	Satisfied	13	10	0.58	9	8	10	10	0.25	0.65	0.47
	Dissatisfied	3	3		2	2	3	0			
	No opinion	3	0		3	0	1	0			
Colour											
What do you think of the colour of the implant tooth?	Satisfied	16	11	1.00	11	8	10	7	0.72	0.56	0.72
	Dissatisfied	3	2		3	2	4	3			
	No opinion	0	0		0	0	0	0

**Table 4:** Modified Eastman Esthetic Index Questionnaire. “Complete group” includes all individuals who participated at T0 (baseline). “Subgroup follow-up” only includes individuals who participated both at T0 and in the long-term follow-up (T1)

Questions		Complete group			Subgroup follow-up						
		T0 (*N* = 32)			T0 (*N* = 24)		T1 (*N* = 24)			T0/T1	
		ISC-L	ISC-C	*P* value	ISC-L	ISC-C	ISC-L	ISC-C	*P* value	ISC-L *P* value	ISC-C *P* value
What do you think about the appearance of your teeth?	Very satisfied	5	3	0.97	4	2	1	1	0.63	0.19	0.35
	Satisfied	13	10		10	8	10	7			
	Dissatisfied	1	0		0	0	3	2			
	Very dissatisfied	0	0		0	0	0	0			
	No opinion	0	0		0	0	0	0			
If you look at your teeth and compare them with your whole face, what do you think about the teeth?	The teeth are one of the most beautiful parts of the face	1	1	0.91	1	1	1	0	0.84	**< 0.001**	**0.03**
	The teeth are prettier than the general impression of the face	16	10		13	8	2	4			
	The teeth are uglier than the general impression of the face	2	2		0	1	6	2			
	The teeth are one of the ugliest parts of the face	0	0		0	0	0	0			
	No opinion	0	0		0	0	5	4			
If you compare with friends your own age, how do you think your teeth look?	Among the prettiest	1	4	0.08	0	4	0	0	0.31	**0.001**	**0.001**
	Better looking than average	15	9		13	6	4	3			
	Uglier than average	3	0		1	0	5	4			
	Among the ugliest	0	0		0	0	0	1			
	No opinion	0	0		0	0	5	2			

*ISC-L* implant-supported crown-lateral, *ISC-C* implant-supported crown-canine

### Function

No differences in headache, tooth clinching and grinding or TMJ sounds were reported by patients with an ISC-L or ISC-C (Table 5).

**Table 5:** Results of temporomandibular disorders (TMD) - Anamnestic Health Questionnaire. “Complete group” includes all individuals who participated at T0 (baseline). “Subgroup follow-up” only includes individuals who participated both at T0 and in the long-term follow-up (T1)

How often have you had any of these problems?		Complete group			Subgroup follow-up						
		T0 (*N* = 32)			T0 (*N* = 24)		T1 (*N* = 24)			T0/T1	
		ISC-L	ISC-C	*P* value	ISC-L	ISC-C	ISC-L	ISC-C	*P* value	ISC-L *P* value	ISC-C *P* value
Headache	Never	12	7	0.50	9	5	6	6	0.77	0.37	1.00
	Monthly	6	6		4	5	7	2			
	Weekly	1	0		1	0	1	0			
	Daily	0	0		0	0	0	0			
Tooth clinching and grinding	Never	14	7	0.32	10	6	10	6	0.59	0.56	0.80
	Monthly	4	4		4	3	2	1			
	Weekly	0	0		0	0	0	0			
	Daily	1	2		0	1	2	3			
TMJ Sounds	Never	16	8	0.27	12	7	11	8	0.93	0.77	0.68
	Monthly	2	3		1	2	2	2			
	Weekly	1	0		1	0	0	0			
	Daily	0	2		0	1	1	0			

*ISC-L* implant-supported crown-lateral, *ISC-C* implant-supported crown-canine

### Clinical examination

#### Periodontal health outcome

Bleeding on probing was noticed more often in ISC-C at T1 compared with T0 (*p* = 0.03). At T1, a pocket depth < 3 mm was found more often in ISC-C than in ISC-L (*p* = 0.01). Furthermore, more sites with increased pocket depth of < 3 mm were detected from T0 to T1 in ISC-C (*p* = 0.001). No significant differences were noticed for buccal gingival retraction in either group, either at T0 or T1 (Table 6).

**Table 6:** Comparison of periodontal parameters between ISC-L (Implant-supported crown-lateral) and ISC-C (Implant-supported crown-canine). “Complete group” includes all individuals who participated at T0 (baseline). “Subgroup follow-up” only includes individuals who participated at both T0 and in the long-term follow-up (T1)

Registration		Complete group			Subgroup follow-up						
		T0 (*N* = 32)			T0 (*N* = 24)		T1 (*N* = 24)			T0/T1	
		ISC-L	ISC-C	*P* value	ISC-L	ISC-C	ISC-L	ISC-C	*P* value	ISC-L *P* value	ISC-C *P* value
Plaque:	Yes	1	1	1.00	1	1	1	0	1.00	1.00	1.00
	No	18	12		13	9	13	10			
Bleeding on probing (BoP):	Yes	11	7	1.00	8	5	8	10	1.00	1.00	**0.03**
	No	8	6		6	5	6	0			
Pocket depth < 3 mm:	Yes	0	0	1.00	0	0	2	7	**0.01**	0.48	**0.001**
	No	19	13		14	10	12	3			
Buccal gingival retraction	Yes	2	1	1.00	2	1	4	0	0.11	0.65	1.00
	No	17	12		12	9	10	10			

#### TMD outcome

No differences in clinical signs of TMD; muscle or jaw pain, deviation of mandible during jaw movements or TMJ sounds, were observed, neither at T0 nor at T1 in patients with ISC-L and ISC-C (Table 7).

**Table 7:** Comparison of signs of temporomandibular disorders (TMD) between ISC-L (Implant-supported crown-lateral) and ISC-C (Implant-supported crown-canine). “Complete group” includes all individuals who participated at T0 (baseline). “Subgroup follow-up” only includes individuals who participated both at T0 and in the long-term follow-up (T1)

Signs of TMD		Complete group			Subgroup follow-up						
		T0 (*N* = 32)			T0 (*N* = 24)		T1 (*N* = 24)			T0/T1	
Groups		ISC-L	ISC-C	*P* value	ISC-L	ISC-C	ISC-L	ISC-C	*P* value	ISC-L *P* value	ISC-C *P* value
Muscle/jaw pain on palpation	Yes	6	4	1.00	4	2	1	0	1.00	0.33	0.47
	No	13	9		10	8	13	10			
Deviation of the mandible during opening or closing	Yes	2	1	1.00	0	1	0	0	0	0	1.00
	No	17	12		14	9	14	10			
TMJ Sounds	Yes	3	2	1.00	0	0	1	2	0.55	1.00	0.47
	No	16	11		14	10	13	8			

#### Prosthodontics outcome

A significant interaction between implant crown colour and the position of the implant was found at T0 χ2 (2) = 6.60, (*p* = 0.0390). Operators more likely graded ISC-L as being too dark (35%) and ISC-C as being too light (40%). However, at T1, no significant colour difference was found between the two groups (χ2 (2) = 2.86, (*p* = 0.2749). Comparing the implant crown colour at T0 with T1 yielded no differences, either for ISC-L or ISC-C (*p* = 0.0625, *p* = 0.1250, respectively).

Even though no significant difference was found, operators more often assessed the gingival colour of ISC-L as being too dark compared with ISC-C at T0 (*N* = 57% and 20%, respectively, *p* = 0.1041) as well as at T1 (*N*=50% and 20%, respectively; *p*= 0.2099). No differences were noticed when the gingival colour at T0 was compared with T1 for either ISC-L or ISC-C (*p* = 1.000, *p* = 0.978, respectively).

Despite no differences being found in the papilla between the groups at T0 or T1, operators considered a greater proportion of patients with ISC-C to have a papilla filling up the entire proximal space than patients with ISC-L at T0 (*N* = 80% and 64%, respectively; *p* = 0.2462) and T1 (*N* = 80% and 71 %, respectively; *p* = 0.8053). In addition, no differences were noted comparing the papilla index at T0 with T1 for either ISC-L or ISC-C (*p* = 1.000, *p* = 0.2500, respectively).

## Discussion

The main result of the present study shows that ISC satisfied the patients, both in the lateral and the canine position, as they were equally satisfied with the aesthetic and functional treatment results. Many previous studies have compared implant installation with orthodontic space closure in patients with dental agenesis [4, 12, 13, 17]. However, there are no comparable studies assessing the pros and cons of implant installation in the lateral and canine position in patients with dental agenesis. For this reason, this prospective cohort study is justified with the aim to evaluate longitudinally, both subjectively and objectively, the aesthetic and functional aspects of ISC-L and ISC-C.

Even though no significant difference in the aesthetics of the ISC was noticed between the groups, the patients in each group were more dissatisfied with the aesthetics at T1 than at T0. Patients’ perception of aesthetics may have changed over the years: what seemed to be aesthetically satisfactory eleven years ago may not be considered as good today, which might explain the decreased satisfaction observed at T1 [20–22]. Another explanation could be that the patients had waited a long time to get an ISC, as implants are installed when the growth of the maxilla has stopped, and were longing for a permanent solution [8, 13]. All the participants in our study were old enough to have an accurate appreciation of dental aesthetics without age affecting their perceptive accuracy [23]. Gender differences in the perception of dental aesthetics and the wish to have orthodontic treatment are well known [24–26]. In the current study, most of the patients in the ISC-L group were men, while most of the patients in the ISC-C group were females. Despite the unequal gender distribution, both groups were similarly satisfied with the aesthetic result.

Previous studies comparing space closure with prosthetic replacement have shown that patients with ISCs have worse periodontal conditions than those with orthodontic space closure [17, 27]. The results from the present study showed worse periodontal status in the ISC-C group, as deeper pockets than in ISC-L patients were found at T1. In addition, greater pocket depth and more BoP was found in the ISC-C group at T1 compared with T0, while no differences were seen among the ISC-L patients. Plaque presence did not differ between the two groups; however, one ISC plaque recording does not reflect the patient’s long-term oral hygiene [28]. Nevertheless, greater pocket depths and more BoP among patients with ISC-C show that these teeth are more difficult to clean. More plaque accumulation among ISC patients has been reported [17, 29]; however, whether the location in the dental arch matters has not been assessed as far as we know. Moreover, the results at T0 demonstrated that even though no significance was seen, more patients with ISC-C than with ISC-L had a papilla that filled up the entire proximal space. The papilla index might be affected by the periodontal status, as poor periodontal health can cause swelling of the gingiva, which can fill up the approximal space to a greater extent [30]. Gingival retraction was only recorded in this study on the buccal sites, as it is presumed to influence the aesthetic perception. In addition, gingival retraction is more frequently seen on buccal sites than on other surfaces [31]. Only a few patients had buccal gingival recession and no statistically significant difference was found between ISC-L and ISC-C. This finding is in accordance with an earlier study comparing buccal gingival recession in patients with ISC and normal teeth [17].

As mentioned above, regardless of the implant position, patients were similarly satisfied with the shape and the colour of the ISC at T0 and at T1. However, at T0, operators graded the colour of ISC-L as being too dark and ISC-C as being too light. Although no significant difference was found, operators also graded the gingival colour of ISC-L being too dark. It is not surprising that professionals are generally more critical than patients regarding aesthetics and satisfaction [32]. In a submitted but so far unpublished trial, the aesthetic assessment of ISCs by orthodontists and laypersons was compared using photos of the participants in the current study. The results showed that both laypersons and orthodontists rated the aesthetics of ISC-L as better than ISC-C with regard both to colour and crown shape.

No significant subjective or objective difference in TMD symptoms was found between the groups. This finding agrees with earlier studies showing a small influence of occlusal factors on the development of TMD or dysfunction [17, 33–35]. The importance of canine lift that was relevant many years ago does not seem to be important nowadays [17, 36].

All implants in this study remained stable with no signs of peri-implantitis and a 100% survival rate, which is in accordance with other studies reporting good a long-term prognosis for single implants in the maxilla [37–40]. However, there are studies reporting complications, such as infraocclusion, which may become an aesthetic concern over time [39, 41–43]. Unfortunately, this was not assessed in this study but may be an interesting aspect to investigate in future studies.

### Limitations

The small number of participants may affect the generalisability of the results and the loss of patients at the follow-up, which is one of the disadvantages of long-term follow-ups. Moreover, it is generally more difficult to attract young people to spend the time to participate in a study. Therefore, due to the small number of patients, the generalisability of the included cases might not be representative for all cases with implant-supported crowns in the lateral and canine positions.

Another factor that may affect the generalisability of the results is that these implant crowns were made over ten years ago and may differ from the materials used today, which are better with regard both to function and aesthetics [44].

## Conclusion

The results from the PROM and clinical examination show that ISC-L and ISC-C are aesthetically and functionally equivalent in the long term. However, ISC-C are more prone to have a periodontal health impact, such as bleeding on probing and pocket depth < 3 mm, than ISC-L. This highlights the importance of maintaining overall good oral hygiene, especially in the canine region, in the presence of an ISC.

### Supplementary information

ESM 1 Supplemental data (survey) is available online at the website of *Clinical Oral Investigations*.

## Data Availability

The data underlying this article will be shared upon reasonable request to the corresponding author.
