# Supplementary material for: Implant-supported crowns on maxillary laterals and canines—a long-term follow-up of aesthetics and function
Source: Clin Oral Investig. 2023 Nov 9;27(12):7545–55. doi: 10.1007/s00784-023-05344-0 (PMC10713674; doi:10.1007/s00784-023-05344-0)
Supplement: Supplementary file 1 — Supplemental data (survey) is available online at the website of Clinical Oral Investigations. [file 784_2023_5344_MOESM1_ESM.docx]

SUPPLEMENTARY MATERIAL - A

**Modified version of the “Eastman Esthetic index”**

1. Are you satisfied with the aesthetic result?
2. Would you consider going through the treatment one more time?
3. Would you recommend the treatment to others?
4. Do you experience the implant tooth as your own tooth?
5. Do you feel that it is more difficult to take care of the implant tooth than your own teeth?

SUPPLEMENTARY MATERIAL - B

**Subjective assessment of Temporomandibular disorder (TMD)**

Are you aware if you usually…….?

1 = Press the teeth together

2 = Grind your teeth

3 = Press with the tongue against the teeth

4 = Bite the cheek/lips/tongue

5 = Combination of two or more parafunctions
